# Metabolomic Profiling of Cellular Responses to Carvedilol Enantiomers in Vascular Smooth Muscle Cells

**DOI:** 10.1371/journal.pone.0015441

**Published:** 2010-11-24

**Authors:** Mingxuan Wang, Jing Bai, Wei Ning Chen, Chi Bun Ching

**Affiliations:** School of Chemical and Biomedical Engineering, College of Engineering, Nanyang Technological University, Singapore, Singapore; Istituto Dermopatico dell'Immacolata, Italy

## Abstract

Carvedilol is a non-selective *β*-blocker indicated in the treatment of hypertension and heart failure. Although the differential pharmacological effects of individual Carvedilol enantiomer is supported by preceding studies, the cellular response to each enantiomer is not well understood. Here we report the use of GC-MS metabolomic profiling to study the effects of Carvedilol enantiomers on vascular smooth muscle cells (A7r5) and to shed new light on molecular events underlying Carvedilol treatment. The metabolic analysis revealed alternations in the levels of 8 intracellular metabolites and 5 secreted metabolites in A7r5 cells incubated separately with *S*- and *R*-Carvedilol. Principal component analysis of the metabolite data demonstrated the characteristic metabolic signatures in *S*- and *R*-Carvedilol-treated cells. A panel of metabolites, including L-serine, L-threonine, 5-oxoproline, myristic acid, palmitic acid and inositol are closely correlated to the vascular smooth muscle contraction. Our findings reveal the differentiating metabolites for A7r5 cells incubated with individual enantiomer of Carvedilol, which opens new perspectives to employ metabolic profiling platform to study chiral drug-cell interactions and aid their incorporation into future improvement of *β*-blocker therapy.

## Introduction

*β*-blockers, also known as *β*-adrenergic receptor antagonists, are a class of drugs that block catecholamines by binding to *β*-adrenergic receptors. There are three known types of *β*-adrenergic receptors, designated *β*_1_ (located manly in heart and kidneys), *β*_2_ (located mainly in lungs, uterus, liver, gastrointestinal tract, vascular smooth muscle and skeletal muscle) and *β*_3_ (located in fat cells). The *β*-adrenergic receptors are a class of G-protein-coupled receptors characterized by seven transmembrane spanning domains forming a pocket in which agonists and antagonists compete for their binding sites [1]. Carvedilol, a nonselective *β*-antagonist (*β*_1_, *β*_2_), has proven potent antihypertensive activity and also effective in the treatment of coronary artery disease and congestive heart failure [2]. It also possesses calcium channel blocking effect by inhibiting voltage-dependent L-type Ca^2+^ in vascular smooth muscle cells [3]. Carvedilol has an asymmetric carbon atom, which gives rise to *S*- and *R*-enantiomers. The racemic mixture of Carvedilol is administered clinically although it results predominantly from *S*-Carvedilol [4], [5]. Although each enantiomer exhibits differential pharmacological effects, the cellular response to individual enantiomer is not well understood. Since the metabolite concentrations represent sensitive responses of cells to external stimuli [6], [7], the analysis of metabolic profiling of A7r5 cells underlying Carvedilol enantiomers treatment will greatly facilitate the understanding on Carvedilol-cell interactions.

We have previously developed LC-MS/MS-based proteomics system to study the cellular responses to the treatments by different generations of beta-blockers [8], [9], [10]. Our study identified several differentially expressed proteins mediating the vascular smooth muscle contraction and also highlighted the differences associated with the treatment of individual enantiomer. While proteomics data has provided important insights into the active effects of *S*-enantiomers, metabolomics may shed new light on the understanding of drug-cell interactions as it reveals the downstream changes of proteome.

Recent advances of instrumentation and computation has enabled the simultaneous analysis of a large number of metabolites. Gas chromatography coupled with mass spectrometry (GC-MS) has proven to be an effective combination for metabolites identifications and quantifications in mammalian cell lines [11], [12], [13], [14] due to its excellent resolution and sensitivity.

The aim of the current study was to obtain a systematic view of the cellular responses to Carvedilol enantiomers. We wished to identify multiple metabolites that could facilitate the understanding of the *in vitro* actions of Carvedilol and aid their incorporation into future improvement of *β*-blocker therapy. In this study, we employed GC-MS-based metabolomic profiling system to functionally assign Carvedilol enantiomers induced changes in the metabolism of A7r5 cells. This study identifies the differential effects of Carvedilol enantiomers on both intracellular and secreted metabolites including those involved in the vascular smooth muscle contraction pathway. Our results support the fact that *S*-Carvedilol possesses more biological activity. Moreover, our study demonstrates the feasibility of GC-MS-based metabolic profiling as an analytical tool to understand drug-cell interactions.

## Results

### GC-MS profiles depict the intracellular response of A7r5 cells to *S*- and *R*-Carvedilol

**Figure 1:**
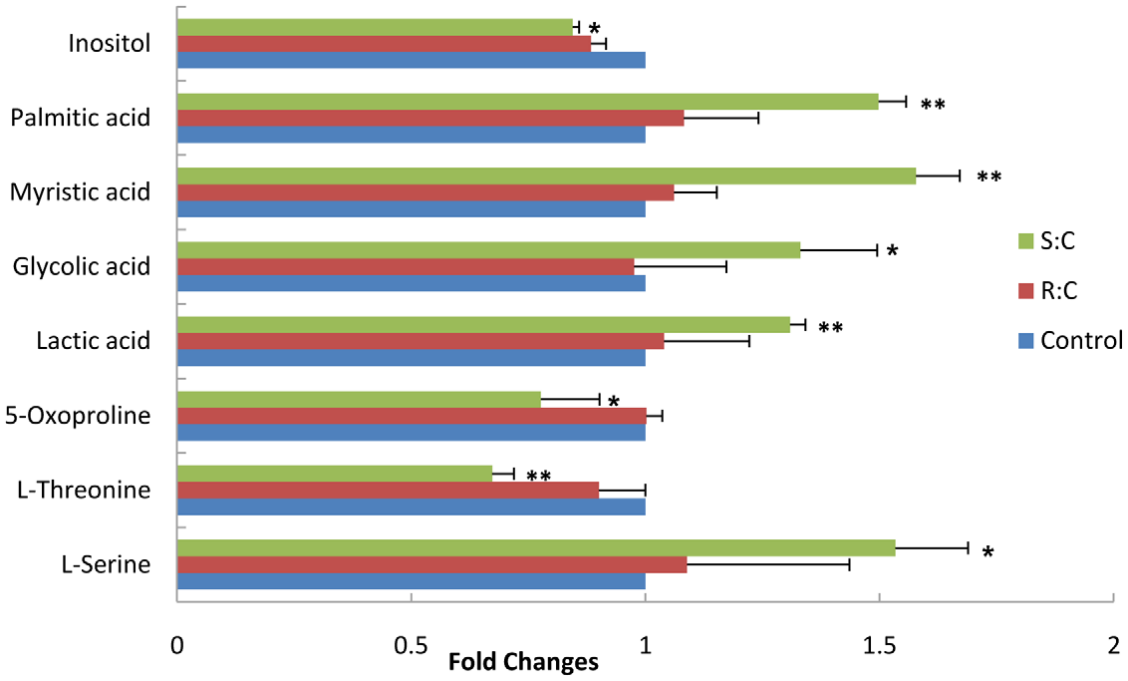
Differential expression levels of intracellular metabolites in *S*- and *R*-Carvedilol-treated cells. The “asterisk” indicated the statistical significance of the metabolite changes by Student's t-test: *, *p*<0.05; **, *p*<0.01.

We performed GC-MS-based metabolomic profiling in A7r5 cells incubated with individual enantiomer of Carvedilol. A total of 32 intracellular metabolites were identified using the NIST library and SHIMADZU GC/MS Metabolite Mass Spectra Database with similarity index above 90%. Out of the 32 metabolites, 8 displayed significantly different levels (*p*<0.05) in A7r5 cells incubated with *S*-Carvedilol, while the changes of most metabolites in *R*-Carvedilol-treated cells are less significant relative to control cells (Figure 1). For example, the levels of palmitic acid and myristic acid displayed up-regulated in *S*-Carvedilol-treated cells while the changes in *R*-Carvedilol-treated cells is not significant compared with control cells.

**Figure 2:**
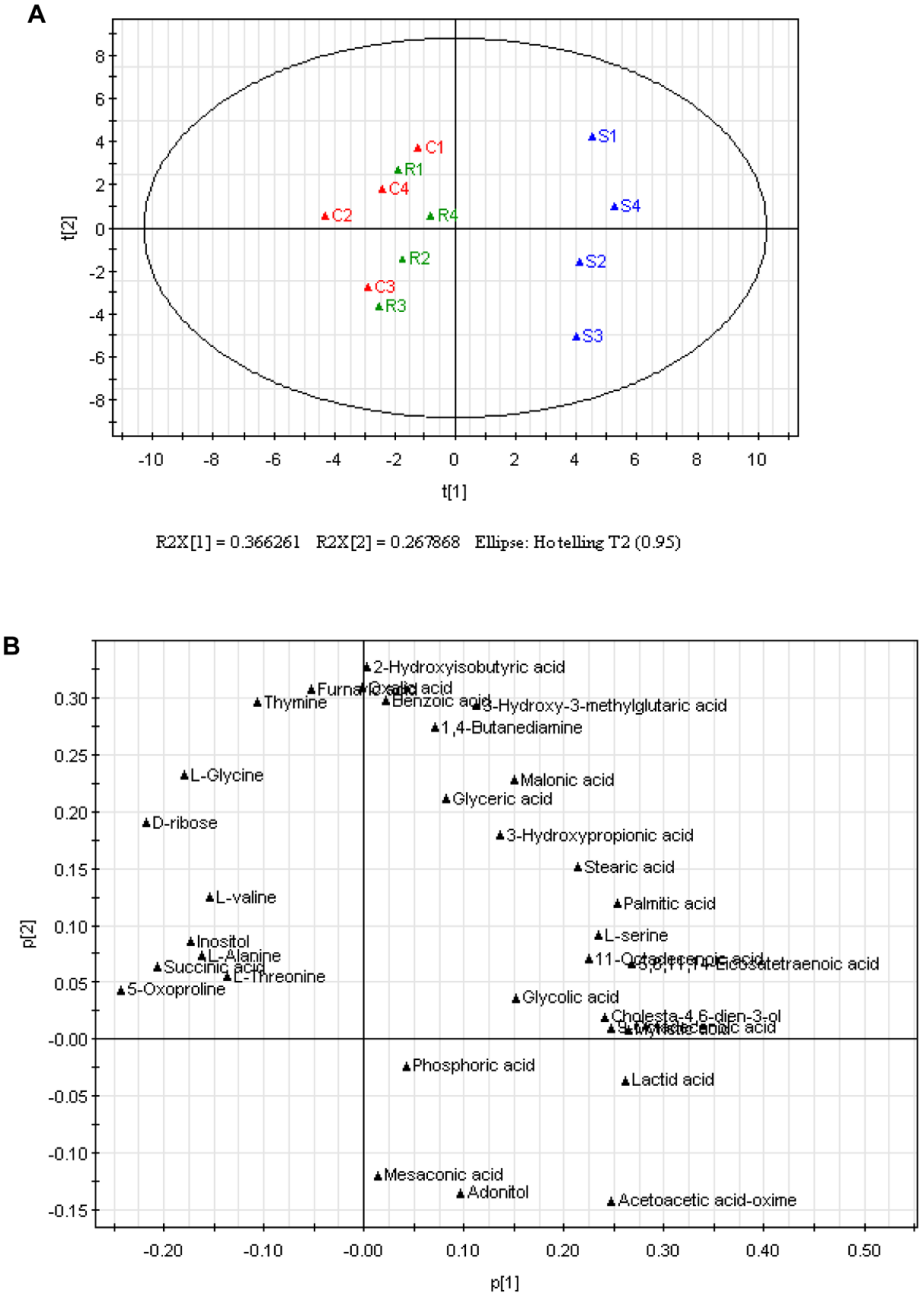
Principal component analysis (PCA) of the GC-MS spectra from intracellular metabolites in A7r5 cells. A) PCA scores plot. Solvent control (red), *S*-Carvedilol (blue) and *R*-Carvedilol (green) are included. B) PCA loading plot.

Multivariate statistical modeling using principal component analysis (PCA) was performed on the GC-MS spectra of four independently grown replicates. PCA has been successfully applied to build classification models from metabolomics data [15], [16], [17], [18]. The scores plot (Figure 2a) resulting from PCA revealed clear discrimination between the intracellular metabolome of A7r5 cells incubated with *S*- and *R*-Carvedilol, indicating that A7r5 cells displayed distinctly metabolic characteristics under individual Carvedilol enantiomer treatment. It was also found out that L-serine, palmitic acid and myristic acid contributed more significantly to distinguishing A7r5 cells incubated with *S*- or *R*-Carvedilol after loadings plot analysis (Figure 2b).

### GC-MS profiles depict the secreted metabolome of A7r5 cells in response to the treatment of *S*- and *R*-Carvedilol

**Figure 3:**
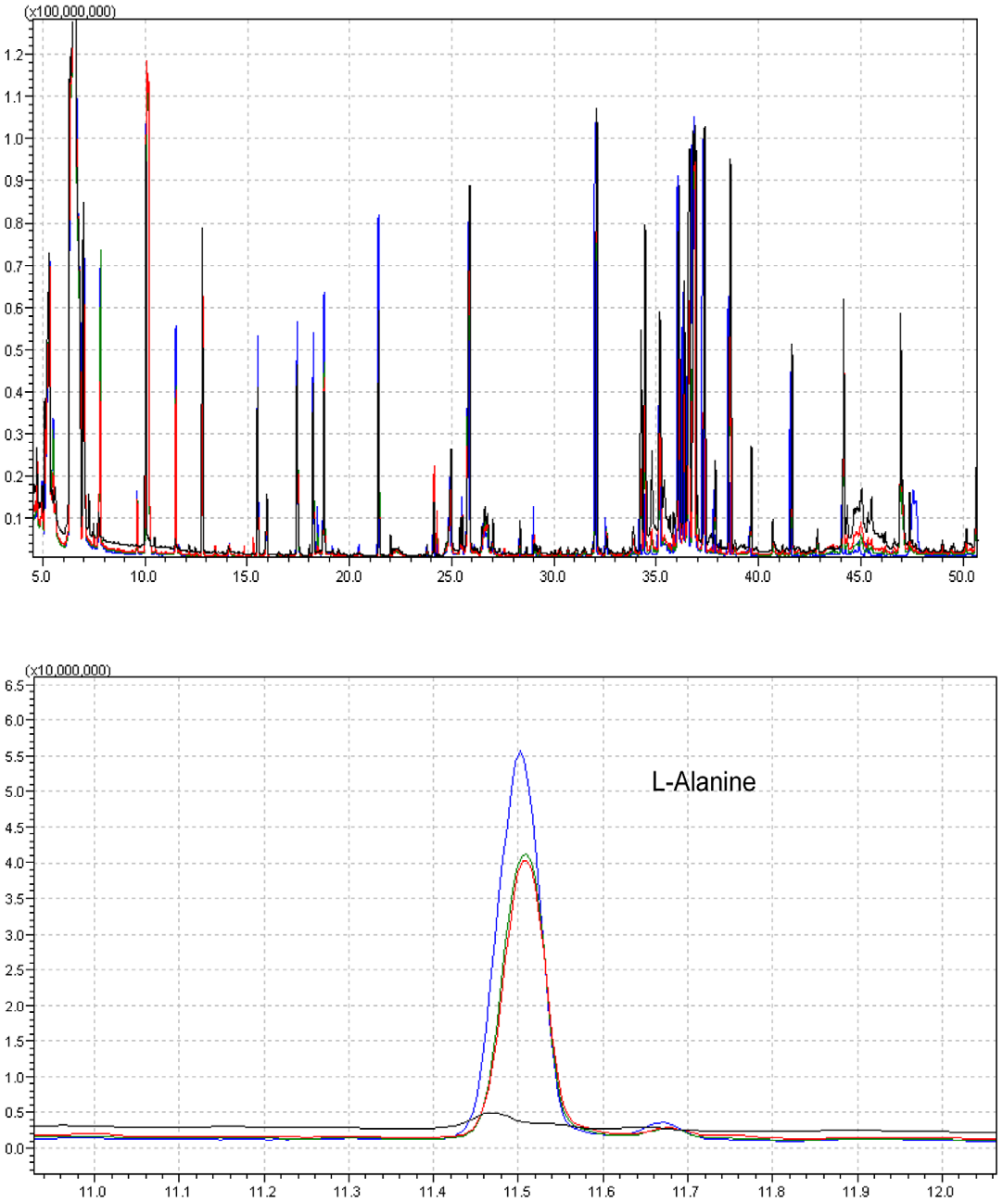
The representive GC-MS spectra of L-Alanine derived from total ion chromatograms. Culture medium (black), untreated cells (red), *R*-Carvedilol-treated cells (green) and *S*-Carevdilol-treated cells (blue).

**Figure 4:**
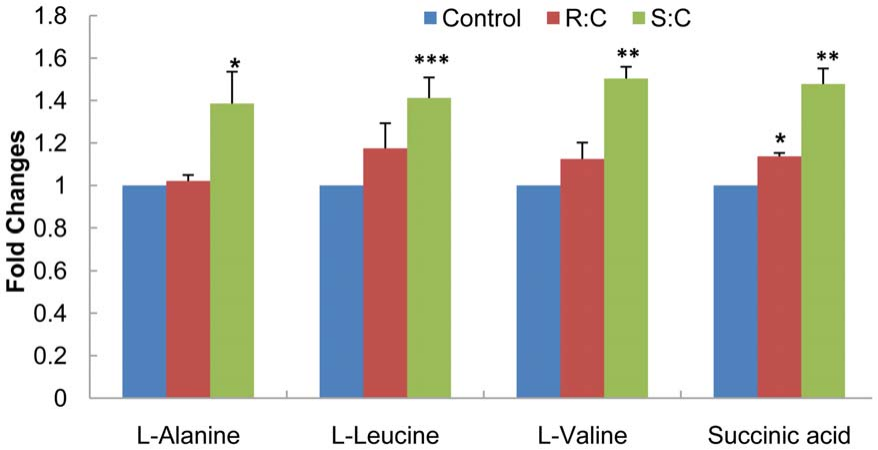
Differential expression levels of secreted metabolites in *S*- and *R*-Carvedilol-treated cells. The “asterisk” indicated the statistical significance of the metabolite changes by Student's t-test: *, *p*<0.05; **, *p*<0.01; ***, *p*<0.001.

**Figure 5:**
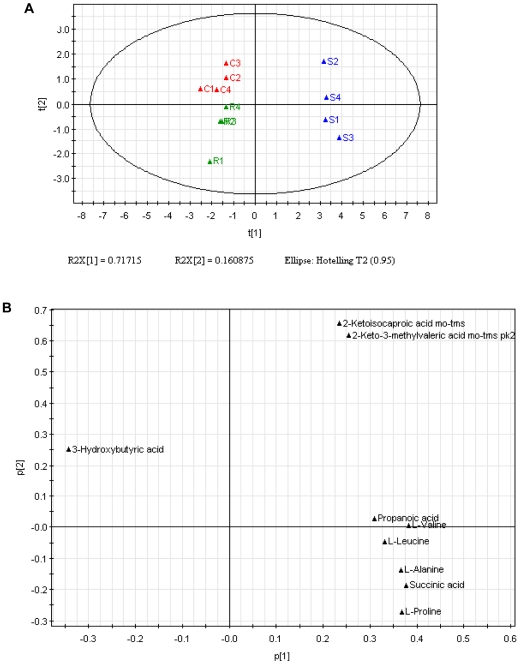
Principal component analysis (PCA) of the GC-MS spectra from secreted metabolites in A7r5 cells. A) PCA scores plot. Solvent control (red), *S*-Carvedilol (blue) and *R*-Carvedilol (green) are included. B) PCA loading plot.

The secreted metabolites in culture medium from A7r5 cells were analysed using GC-MS system. Analysis detected 9 secreted metabolites. A representive GC-MS total ion chromatograms is shown in Figure 3 and the up-regulated metabolite L-Alanine was observed in *S*-Carvedilol-treated cells. The culture medium incubated at the same conditions was set as negative control. From four independent exepriments, 4 metabolites were indentified as significantly changes between samples incubated with *S*-Carevdilol and control, while the changes in *R*-Carevdilol-treated cells is less significant (Figure 4). PCA was performed on the GC-MS spectra of four biological replicates from A7r5 cells incubated with control, *S*- and *R*-Carvedilol. The PCA scores plot shown in Figure 5a demonstrates that exposure of A7r5 cells to *S*- or *R*-Carvedilol caused distinct changes in the metabolome. Loadings plot identified L-alanine, L-leucine, L-valine and succinic acid as the major discriminators between *S*- and *R*-Carvedilol-treated cells (Figure 5b).

### Intracellular Calcium concentration variations in response to Carvedilol enantiomers treatment

To investigate the calcium inhibition effect of each Carvedilol enantiomer, the intracellular Ca^2+^ concentration was measured. The results indicated that the intracellular concentration in *S*-Carvedilol-treated A7r5 cells was lower than that in control cells (S:C, 0.848±0.018, *p*<0.05), while the variations were less significant in *R*-Carvedilol-treated cells (R:C, 0.935±0.012, *p*<0.05). Student's t-test was performed to assess the significance of the variance.

## Discussion

In pharmacology, the importance of drug chirality and its role in explaining the dramatic differences of individual enantiomers in biological activities is increasingly appreciated [19]. Our previous studies investigated the intracellular proteome in A7r5 cells incubated with individual enantiomers of *β*-blockers and identified differentially expressed proteins mediating the vascular smooth muscle contraction [8], [9], [10]. In the current study, we have applied GC-MS-based metabolomic profiling approach to investigate the cellular response to the treatment of Carvedilol enantiomers. We wished to investigate whether a metabolomic approach could identify signatures associated with *S*- and *R*-Carvedilol treatment and whether these signatures could shed new light into the *in vitro* actions of Carvedilol. To our knowledge, this is the first study to apply a metabolic profiling system to characterize chiral drug-cell interactions. The GC-MS-based metabolic profiling platform paired with multivariate statistical method (PCA) has enabled us to highlight the distinct cellular response to individual enantiomers of Carvedilol and several metabolites altered in the treatment of *S*-Carvedilol.

To obtain the information about the metabolic variations underlying the treatment of Carvedilol enantiomers, we first analysed the intracellular metabolome in A7r5 cells. From four independent experiments, 8 metabolites were consistently identified with statistically significant changes and closely relevant with the cell functions associated with the treatment of *S*-Carvedilol and will be discussed as follows.

**Figure 6:**
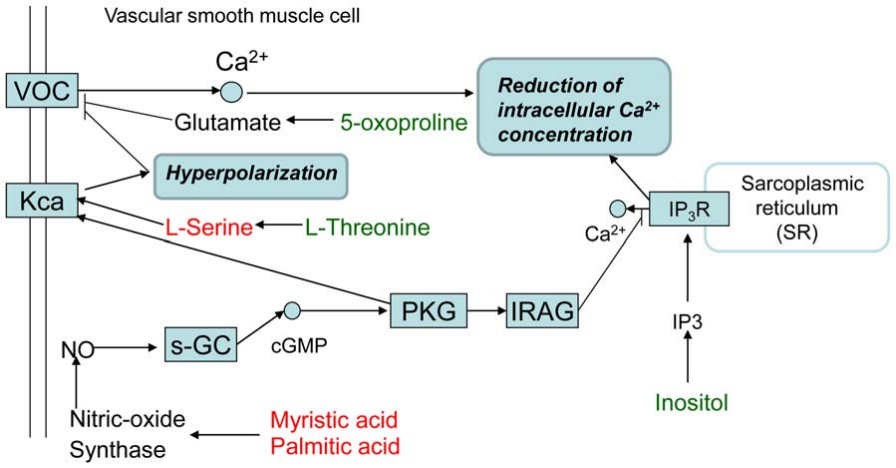
Schematic representation of the vascular smooth muscle relaxation pathway showing the most relevant metabolic changes inudced by *S*-Carvedilol. Metabolites in red/green display increased/decreased concentrations upon *S*-Carvedilol treatment.

The intracellular Ca^2+^ has been a central player in regulating vascular smooth muscle contraction and relaxation [20], [21], [22], [23]. A decreased of intracellular Ca^2+^ concentration will elicit relaxation. The previous study has suggested that Carvedilol exhibited the inhibitory effects on Ca^2+^ mobilization and this unique action may contribute to its antihypertensive effect [3]. Using KEGG database and literature mining, we have linked the intracellular metabolic variations to the vascular smooth muscle relaxation pathway (Figure 6). Changes in amino acid levels were variable. L-serine, a precursor of central neurotransmitters, was found to be up-regulated in cells incubated with *S*-Carvedilol compared with control cells and those incubated with *R*-Carvedilol. The up-regulated L-Serine has been reported to exhibit antihypertensive effect by evoking vasodilatation in vascular smooth muscles cells [24]. The vasodilatation effect is mediated by activating Ca^2+^-activated K^+^ channel [25], which induces the hyperpolarization in vascular smooth muscle cells and promotes the closure of voltage-operaetd calcium channels (VOC). The inactiviated vascular smooth muscle VOC thus reduces Ca^2+^ influx, which results in the Ca^2+^ reduction and subsequent relaxation or vasodilatation. In contrast, levels of L-threonine and 5-oxoproline were reduced in *S*-Carvedilol-treated cells. L-threonine is an essential amino acid that could be used for serine biosynthesis [26]. 5-oxoproline is converted into glutamate by 5-oxoprolinase [27]. Glutamate activates glutamate receptor that inhibits calcium channel, which induces muscle relaxation. Despite great effort, we were not able to identify glutamate in this study.

The fatty acids level treaded toward increase in cells incubated with *S*-Carvedilol. The up-regulated myristic acid and palmitic acid have been reported to stimulate nitric-oxide Synthase, which in turn actives the production of nitric oxide (NO) [28]. In the cardiovascular system, NO plays an essential role in hemodynamics and controlling vascular remodeling [29], [30], [31]. For example, in vascular smooth muscles cells NO stimulates soluble guanylyl cyclase (s-GC) to generate the second messenger cGMP, which results in activation of cGMP-dependent protein kinase G (PKG) [32], [33]. Inositol trisphosphate receptor (IP_3_R) is a membrane glycoprotein complex acting as Ca^2+^ release channel activated by the second messenger inositol trisphosphate (IP_3_). The activated PKG is recruited by the IP_3_R-associated cGMP kinase substrate (IRAG), resulting in IP_3_R phosphorylation and inhibition of Ca^2+^ release from endoplasmic reticulum (ER) [34]. The reduced Ca^2+^ concentration in vascular smooth muscle cells induced by increased NO production thus results in muscle relaxation. The Ca^2+^ concentration measurement assay was conducted to prove our hypothesis and the results indicated that the Ca^2+^ concentration in *S*-Carvedilol-treated cells is down-regulated compared with control cells and those incubated with *R*-Carvedilol. In addition, our observation of a decreased level of inositol in cells incubated with *S*-Carvedilol may be due to the decrease of IP_3_ caused by the phosphorylation of IP_3_R.

Lactic acid, an end product of glycolysis, was up-regulated in cells incubated with *S*-Carvedilol. A higher concentration of lactic acid is produced in vascular smooth muscle cells when faced with low levels of oxygen. Intaking of Carvedilol may cause some common side effects in clinic such as shortness of breath, dizziness [35], [36]. Our observation of increased level of lactic acid could be related with the side effects induced by *S*-Carvedilol. Glycolic acid is the smallest α-hydroxy acid which is able to exfoliate dead skin cells and encourage cell regeneration. The function of glycolic acid in vascular smooth muscle cells in unclear. The increased glycolic acid could be possibly involved in the inhibition of vascular smooth muscle thickening that spontaneously happens in hypertensive rats [37].

Metabolites secretion by a cell reflects the biochemical response to external stimuli. We have previously investigated the secreted proteome in A7r5 cells incubated with *β*-blockers and a common secreted protein T-kininogen was identified as a potential drug marker [38], [39]. Since the metabolites are the end products of cellular regulatory processes, the quantitative analysis of the secreted metabolites in A7r5 cells upon Carvedilol enantiomers treatment should provide us a complementary understanding on overall cellular response. A panel of 4 metabolites (L-alanine, L-leucine, L-valine, succinic acid) showed up-regulated in cells incubated with *S*-Carvedilol compared with control cells and those incubated with *R*-Carvedilol. L-leucine and L-valine are essential amino acids with three branched-chains, which could be used as an energy source by muscle tissue. L-alanine can be produced from branched chain amino acids, such as L-leucine and L-valine. The possible reason that the high secreted levels of these amino acids is to keep them in balance in the more relaxed cells. PCA analysis demonstrates the characteristic signatures of cells incubated with individual enantiomers of Carvedilol. Succinic acid secretion was strongly enhanced with *S*-Carvedilol treatment, which may result from the inactivation of succinate dehydrogenase. The highly produced NO in *S*-Carvedilol-treated cells may selectively inhibit succinate dehydrogenase activity [40]. Since the secreted metabolites variations in a cell reflect its internal metabolic state, the distinct secretion profiles further confirm the differences of intracellular metabolome of A7r5 cells in response to the *S*- and *R*-Carvedilol treatment. However, no specific production or secretion pathways were found to connect secreted metabolic variations to the changes in intracellular metabolic states due to the limited secreted metabolites seen. The possible explanations for this: 1) Metabolites are very instable and degraded rapidly in culture medium. 2) Partial of the secreted metabolites may not be amenable to derivatization for GC-MS analysis. The complementary analytical technologies (for example, LC-MS, NMR) without such derivatization may become alternative ways to investigate the secreted metabolome in A7r5 cells. This should be dressed in our further study.

In conclusion, our results highlight the potential of GC-MS-based metabolic profiling of A7r5 cells in response to Carvedilol enantiomers treatment. The cells incubated with individual enantiomers demonstrate characteristic metabolic signatures, which is consistant with the fact that each enantiomer exhibits differential biological activities. Specifically, the metabolic changes observed in A7r5 cells in response to *S*-Carvedilol treatment were closely involved in the vascular smooth muscle relaxation pathway. These findings enhance our understanding of Carvedilol-cell interactions, which may in turn aid their incorporation into future improvement of *β*-blocker therapy.

## Materials and Methods

### GC-MS sample preparation

Vascular smooth muscle cell line (A7r5) was obtained from American Type Culture Collection (ATCC) and cultured in Dulbecco's Modified Eagle's Medium (DMEM) supplemented with 10% fetal bovine serum (FBS), 100 U/mL penicillin and 100 U/mL streptomycin (Life Technologies). Cells were maintained at 37°C in an atmosphere of 5% CO_2_ with the medium replaced every 24 hours.

1.5×10^7^ cells were treated with either *S*- or *R*-Carvedilol at a concentration of 5 µM due to MTT reduction analysis [10]. Cells treated in parallel with equal amounts of dimethyl sulfoxide (DMSO) were used as control. After 48 hours, culture medium was collected, filtered through a 0.2 µm filter (WHATMAN), and immediately frozen in liquid nitrogen. Cells were washed with cold PBS twice, harvested and snapped frozen in liquid nitrogen. Carvedilol enantiomers were previously separated from their racemic form using High Performance Liquid Chromatography (HPLC) in our lab [10]. Four independent experiments were conducted.

The extraction of intracellular metabolites from A7r5 cells was performed using a modified Mainak Mal [41] and Hindrik Mulder [11] procedure. Cell pellets were dissolved in a mixture of chloroform/methanol/water in ratio of 2∶5∶2 (v/v/v). Ribitol (2 mg/mL dissolved in water, 10 µL) was added into the extraction solvent as internal standard to correct for metabolites losses during sample preparation. The samples were ultra-sonicated in an ice bath ultra-sonicator for 30 min and subsequently centrifuged at 18000 *g* for 10 mins at 4°C. 0.8 mL supernatant was collected and evaporated to complete dryness overnight using a TurboVap® LV Concentration Workstation. The culture medium samples were prepared by spiking 500 µL of each culture medium with 10 µL internal standard solution (ribitol, 2 mg/mL dissolved in water) and lyophilised. Immediately prior to GC-MS analysis, derivatization was performed in two steps: Firstly, methoximation was carried out by dissolving the sample in 50 µL of 20 mg/mL solution of methoxyamine hydrochloride in pyridine to protect the carbonyls. The incubation was kept at 37°C for 60 min. Silylation was then carried out by adding 100 µL of N-methyl-N-(trimethylsilyl)-trifluoroacetamide (MSTFA) with 1% trimethylchlorosilane (TMCS) to each sample for 30 min at 70°C. After incubation, samples were shaken for 2 hours at room temperature and then transferred to vials for GC-MS analysis.

### GC-MS analysis

All samples were analysed using a Shimadzu QP2010Plus GC-MS system (Shimadzu, Kyoto, Japan) equipped with a DB-5 fused-silica capillary column (30 m×250 µm i.d.; film thickness: 0.25 µm; Agilent J&W Scientific, Folsom, CA, USA). Each 1 µL of the derivatised samples was injected into GC-MS system by Shimadzu AOC-20i+s auto-sampler in the splitless mode. The solvent cutoff time was set at 3.5 min. Helium was used as carrier gas at 1.1 mL/min. The injection and ion source temperatures were kept at 280 and 200°C, respectively. The oven temperature was kept at 100°C for 4 min and increased at 4°C/min to 320°C where it remained for 1.56 min. Detection was implemented in electron impact (EI) ionization mode at 70eV. Mass spectra were recorded from 35 to 600*m/z* with a scan time of 0.3 s. Chromatogram acquisition and mass spectra identification were processed using the Shimadzu GCMSsolution (version 2.5) software. To allow normalization of retention times, a retention index (RI) solution (C-10 to C-40) was injected into the GC-MS system and performed by AART (Automatic Adjustment of Retention Time) function of GCMSsolution software. All chromatograms were subjected to noise reduction and baseline correction prior to peak area integration. Chromatographic peak deconvolution was performed using the following parameters: peaks width = 2 s, smoothing = 3. For those peaks detected, the peak area was calculated and normalized using the internal standard generating a response ratio. Chemical identification of detected metabolite peaks was preformed by searching NIST mass spectral library and SHIMADZU GC-MS Metabolite Mass Spectra Database (Release 1.0). Identification was regarded as preliminary for spectra matched in the NIST library with a similarity above 90%. Identifications were regarded as definitive if the mass spectrum and retention index matched those of a metabolite present in the SHIMADZU GC-MS Metabolite Mass Spectra Database, which was prepared by analysis of authentic chemical standards on the instrument employed in this study. All samples were analysed within 24 hours in a random order.

### Intracellular Ca^2+^ concentration Measurements

The intracellular Ca^2+^ concentration was measured using a Fluo-4 NW Calcium Assay Kit (Invitrogen, Eugene, OR, USA) according to the manufacturer's protocol. Briefly, each type of A7r5 cells was incubated at 37°C for 30 min in the Fluo-4 dye solution dissolved in assay buffer (Component C, Invitrogen) in a 96-well plate. Following incubation, cells were equilibrated at room temperature for an additional 30 min. The assay was performed using a fluorometer (Tecan) at 494 nm for excitation and 516 nm for emission.

### Statistical analysis

The significance of intracellular and secreted metabolite level changes from A7r5 cells in response to Carvedilol enantiomers treatment was calculated using the Student's t-test, where all detected metabolites were tested and a threshold of *p*<0.05 was used all this study. Principle component analysis (PCA) was performed to determine whether intracellular and secreted patterns of metabolite changes in A7r5 cells under treatment of *S*- and *R*-Carvedilol, in addition to the altered levels of individual metabolites. PCA analysis is an unsupervised clustering method aiming at summarizing data with reduced dimensions. In this study, four biological replicates were used to perform PCA analysis for each sample using the SIMCA-P+ 12 (Umetrics, Umea, Sweden) software.
